# Unprecedented Mechanochemical Synthesis and Heterogenization of a C-Scorpionate Au(III) Catalyst for Microwave-Assisted Biomass Valorization

**DOI:** 10.3390/nano12030362

**Published:** 2022-01-23

**Authors:** Inês A. S. Matias, Pablo G. Selfa, Ana M. Ferraria, Ana M. Botelho do Rego, Maximilian N. Kopylovich, Ana P. C. Ribeiro, Luísa M. D. R. S. Martins

**Affiliations:** 1 Centro de Química Estrutural, Institute of Molecular Sciences, and Departamento de Engenharia Química, Instituto Superior Técnico, Universidade de Lisboa, 1049-001 Lisboa, Portugal; ines.matias@tecnico.ulisboa.pt (I.A.S.M.); pablogselfa@tecnico.ulisboa.pt (P.G.S.); maximilian.kopylovich@tecnico.ulisboa.pt (M.N.K.); 2 iBB—Institute for Bioengineering and Biosciences and Departamento de Engenharia Química, Instituto Superior Técnico, Universidade de Lisboa, Av. Rovisco Pais, 1049-001 Lisboa, Portugal; ana.ferraria@tecnico.ulisboa.pt (A.M.F.); amrego@tecnico.ulisboa.pt (A.M.B.d.R.); 3 Associate Laboratory i4HB, Institute for Health and Bioeconomy at Instituto Superior Técnico, Universidade de Lisboa, Av. Rovisco Pais, 1049-001 Lisboa, Portugal

**Keywords:** catalysis, mechanochemistry, C-scorpionate complex, microwaves, glycerol, furfural, graphene, heterogenization, sustainable process

## Abstract

The transformation of biomass, a carbon resource presenting a huge potential to produce valuable chemicals, requires the search for sustainable catalytic routes. This work proposes the microwave-assisted oxidation of biomass -derived substrates, such as glycerol and the furfural derivatives 5-(hydroxymethyl)furfural (HMF) and 5-hydroxymethyl-2-furancarboxylic acid (HFCA), using the C-scorpionate dichloro-gold(III) complex [AuCl_2_(κ^2^-Tpm)]Cl (Tpm = HCpz_3_; pz = pyrazol-1-yl) as a catalyst, as prepared and supported on graphene, in solvent-free conditions. The unprecedented application of a mechanochemical procedure (in a planetary ball mill, in solid state) to synthesize a C-scorpionate complex, the [AuCl_2_(κ^2^-Tpm)]Cl, is disclosed. The immobilization of [AuCl_2_(κ^2^-Tpm)]Cl on graphene was performed using different methods, including some (e.g., microwave irradiation and liquid assisted grinding) for the first time. The structural properties and the performance of the prepared catalytic materials are presented and discussed.

## 1. Introduction

Global warming is presented as one of humanity’s major concerns, with the depletion of fossil fuel resources, the continuous generation of greenhouse gas (GHG) emissions, and the dramatic increase of the world’s population [1]. A significant part of these GHG emissions is related to anthropogenic activities (in Europe, in 2018, the total waste generated by all economic activities and households amounted to 2317 million tons [2]).

A new sustainable alternative to traditional petrochemical feedstock processes, leading to a competitive, energy-efficient, and low carbon future, can be attained by using organic wastes as feedstock in chemical industry. Thus, biomass is potentially one of the most promising resources to produce biofuel and fine chemicals.

**Scheme 1:**
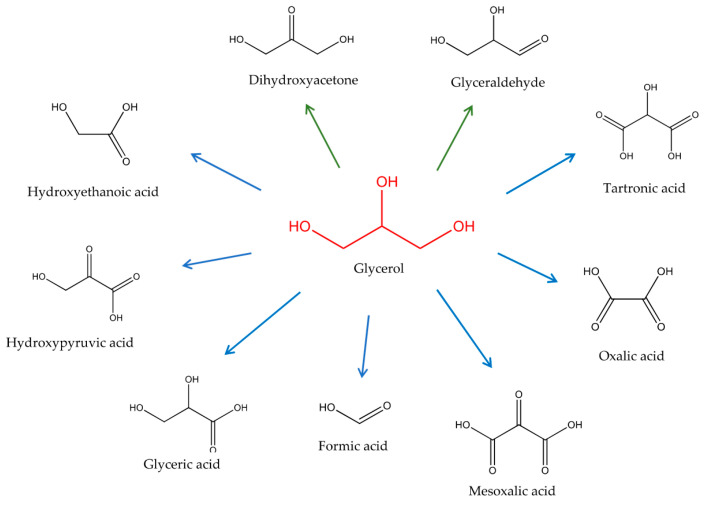
Products resulting from glycerol oxidation [4,5].

Glycerol, an important by-product of biodiesel production, is formed in significant amounts by transesterification of triglycerides with methanol and can be used as feedstock to produce high value-added products [3]. An effective utilization of glycerol by its efficient conversion into value-added bio-based commodities (Scheme 1) would have a very positive effect on biodiesel production economics, with its demand forecast to increase in the following years [4,5]. It also offers a great alternative to reduce fossil feedstocks’ dependence, decrease the CO_2_ emissions in this sector, and boost cleaner, more efficient, modern, and better-oriented industries that are able to satisfy the increasing demand for eco-friendly, safer, and healthier products.

**Scheme 2:**
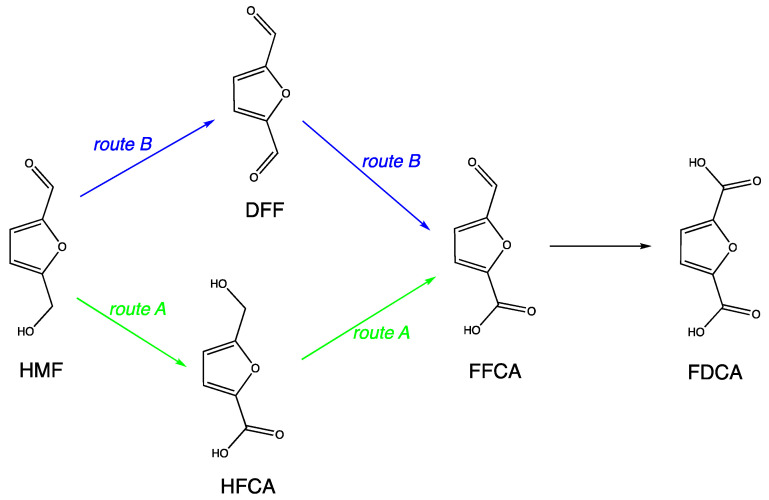
Reaction pathways of 5-(hydroxymethyl)furfural (HMF) oxidation to produce 2,5-furandicarboxylic acid (FDCA) [6]. Route A: aldehyde group oxidation to 5-hydroxymethyl-2-furan carboxylic acid (HFCA). Route B: alcoholic group oxidation to 2,5-furan dicarboxaldehyde (DFF).

In addition, furfural derivatives, produced by glucose or fructose dehydration, can be key reagents to produce many important products as polymers, pharmaceuticals, solvents, or fuels [5]. For example, 2,5-furandicarboxylic acid (FDCA) has been considered as an excellent precursor, instead of petroleum-derived terephthalate acid, for producing green polymers such as polyethylene 2,5-furandicarboxylate (PEF). In a recent attempt, biopolymers have been successfully produced from bio-based ethylene glycol and FDCA, [6] which can be obtained from the catalytic oxidation of 5-(hydroxymethyl)furfural (HMF) via formation of 5-formyl-2-furancarboxylic acid (FFCA), as depicted in Scheme 2.

Suitable catalysts for HMF oxidation include oxides of noble or transition metals, but the high cost and poor recycling of the former and the low FDCA yield of the latter are significant hindrances to their commercial acceptance.

It is worth mentioning that only chemical innovations conducted in a sustainable way can allow progress in achieving the United Nations Sustainable Development Goals (SDGs) [7]. Thus, the search for sustainable catalytic synthetic processes from biomass resources is mandatory and urgent.

Herein, we present the microwave-assisted peroxidative oxidations of glycerol to dihydroxyacetone and the furfural derivatives 5-(hydroxymethyl)furfural (HMF) and 5-hydroxymethyl-2-furancarboxylic acid (HFCA) to 2,5-furandicarbaldehyde and 5-formylfuran-2-carboxylic acid (FFCA), respectively. Microwave irradiation has proved to be effective in saving energy as reactants are heated by transferring electromagnetic radiation (low-frequency oscillating electric and magnetic fields) directly into the molecules, while leaving the reactor unheated [8].

**Figure 1:**
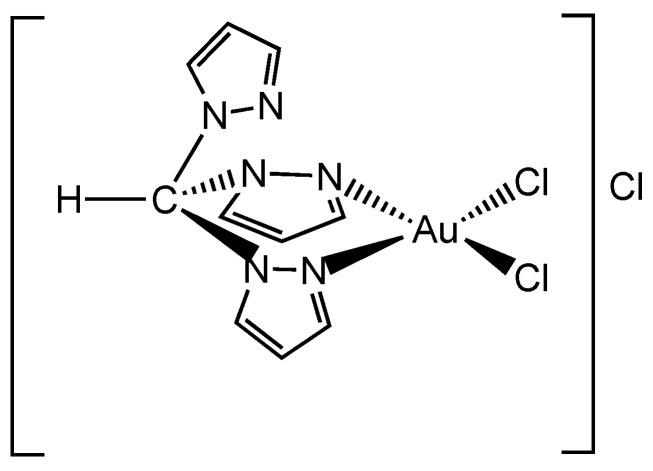
Structure of hydrotris(1*H*-pyrazol-1-yl)methane dichloro-gold(III) complex [AuCl_2_(κ^2^-Tpm)]Cl (Tpm = HCpz_3_; pz = pyrazol-1-yl) [9,10].

Further, a C-scorpionate complex, the hydrotris(1*H*-pyrazol-1-yl)methane dichloro-gold(III) complex [AuCl_2_(κ^2^-Tpm)]Cl (Tpm = HCpz_3_; pz = pyrazol-1-yl, Figure 1), was chosen as a catalyst in view of its synthetic simplicity and ability to efficiently catalyze challenging oxidation reactions such as the oxidation of non-activated cycloalkanes [9]. The designation “scorpionate” is attributable to Trofimenko, who discovered the class ligands, as he thought they resembled the way a scorpion attacks its prey. There is coordination to the metal center in a bi- or tri-dentate way, with the analogy of using only the claws or also the tail to hold the prey (meaning the metal).

In fact, C-scorpionate metal complexes are known as one of the few mononuclear types of metal compounds able to catalyze the oxidation of alkanes [11,12,13,14] and hydrocarbons such as xylene [15], secondary alcohols [16], ketones [17], and alkenes [18]. Moreover, they can be used as homo- or heterogeneous catalysts and in recent years, their catalytic applications have experienced significant advances [10,13,14,19,20,21,22]. Thus, herein, an improved catalytic material was designed, aimed at mixing the benefits of homo- and heterogeneous catalysts, by immobilizing the C-scorpionate gold(III) catalyst onto a solid support. Graphene was chosen among the carbon-based materials [23] due to its unique properties (e.g., intrinsic carrier mobility at room temperature, mechanical strength, chemical and thermal stability, etc.) which may play a role during the catalytic process. Although not catalytic active for this reaction, graphene acts as a platform for anchoring the molecular complex, thus enhancing its catalytic activity by avoiding aggregation and facilitating the interaction between substrates and catalytic active molecular species.

On the other hand, the mechanochemical route was followed as an alternative to achieve the goal of green synthesis [24]. This enabling technology is characterized by mechanical grinding of reactants and induces chemical reactivity by mechanical force (e.g., by compression, shear, or friction). It provides green advantages since it is solvent-free, enables short reaction times, high efficiency, and unique reactivity. As stated by Sheldon [25], “The best solvent is no solvent…”, and herein the neat (dry) grinding synthetic strategy is followed to prepare [AuCl_2_(κ^2^-Tpm)]Cl. To the best of our knowledge, there is no report on the effect of a planetary ball mill under ambient conditions to produce C-scorpionate complexes compared to the liquid phase chemical route. Moreover, it is the first time that the heterogenization of C-scorpionate complexes on C-materials is performed by a mechanochemical procedure.

## 2. Materials and Methods

All operations were carried out in open air, unless stated otherwise. All reagents and solvents were purchased from commercial sources (Sigma-Aldrich, Munich, Germany) and used as received. Hydrotris(1*H*-pyrazol-1-yl)methane, Tpm, HC(pz)_3_ (pz = pyrazoly-1-yl) was prepared through a literature method [26,27] and characterized by conventional techniques.

Fourier transform infrared spectroscopy (FTIR) analyses were performed in KBr pellets (4000–400 cm^−1^) or CsI (400–180 cm^−1^) on a Bruker Vertex 70 Raman/IR spectrometer (Barcelona, Spain) in a range from 4000 to 180 cm^−1^.

Ultraviolet-visible spectroscopy (UV-vis) measurements were recorded on a lambda 35, Perkin Elmer spectrophotometer (Waltham, MA, USA).

Proton nuclear magnetic resonance (^1^H NMR) spectra (chemical shifts δ expressed in ppm relative to SiMe_4_) were obtained at room temperature on a Bruker Advance 300 MHz spectrometer (Gratz, Austria).

The inductively coupled plasma atomic emission spectroscopy (ICP-AES) and C, N, and H elemental analyses were carried out by the Microanalytical Service of the Instituto Superior Técnico.

A scanning electron microscope (SEM) (JEOL 7001F with Oxford light elements EDS detector and EBSD detector, JEOL, Tokyo, Japan) was used to characterize the morphology of the graphene-supported C-scorpionate gold(III) complexes.

X-ray Photoelectron Spectroscopy (XPS) analyses were performed using a XSAM800 spectrometer (KRATOS Analytical, Manchester, UK) with non-monochromatic Mg K_α_ X-radiation (hν = 1253.6 eV) at TOA = 45°. The binding energy (BE) of reference used to correct the charge shift was the BE of sp^2^ carbon, typically centered at 284.7 eV. The quantification was performed using the sensitivity factors from the library of Vision 2 for Windows, Version 2.2.9 from KRATOS (software of spectra acquisition). Other operational conditions and data treatment details are as published elsewhere [28].

Two different ball mill (BM) equipment models were used to synthesize the C-scorpionate Au(III) complex and anchor it on graphene: the Planetary Ball Mill PM 100 and the Emax High Energy Ball Mill (both from Retsch Haan, Germany). Each mill has two stainless steel milling jars rotating around its axis which should be partially filled with milling balls.

Catalytic reactions under microwave (MW) irradiation were performed in a focused Anton Paar Monowave 300 reactor (Anton Paar GmbH, Graz, Austria) fitted with an IR temperature detector and a rotational system, in a Pyrex cylindrical tube (10 mL or 30 mL capacity).

### 2.1. Synthesis of C-Scorpionate Gold(III) Complex, [AuCl_2_(κ^2^-Tpm)]Cl

For the first time, the C-scorpionate gold(III) complex [AuCl_2_(Tpm)]Cl was prepared by mechanochemical dry synthesis, using a planetary ball mill reactor (see above). The reactants, gold(III) chloride hydrate (0.2–0.5 mmol) and hydrotris(pyrazol-1-yl)methane (HCpz_3_, pz = pyrazol-1-yl; Tpm) (0.2 mmol), were subjected to mechanochemical treatment with stainless steel milling balls (5 mm; 7.7 kg dm^−3^). Different planetary ball mill models (PM 100 and Emax) and reaction conditions—milling time (5–30 min), number of milling balls (1–5), and rotation frequency (100–800 min^−1^)—were tested for the synthesis of [AuCl_2_(κ^2^-Tpm)]Cl.s. The resulting bright yellow powder was collected from the milling jar and vacuum-dried overnight at 60 °C.

**Scheme 3:**
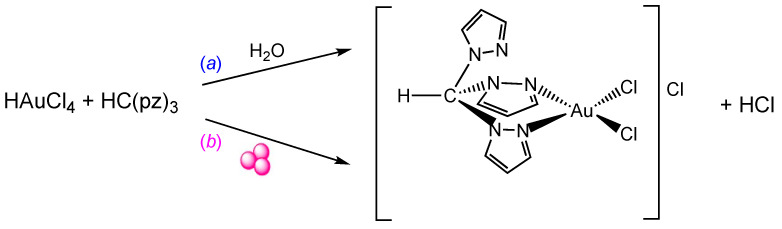
Synthesis of the C-scorpionate gold(III) complex, [AuCl_2_(κ^2^-Tpm)]Cl, in conventional liquid phase (Route a) and by dry mechanochemistry (Route b).

[AuCl_2_(κ^2^-Tpm)]Cl was also synthesized in liquid phase conditions, according to the literature [9], by reacting equimolar amounts (0.2 mmol) of Tpm and gold(III) chloride hydrate, HAuCl_4_·3H_2_O, in distilled water, at room temperature (see Scheme 3 below).

### 2.2. Immobilization of [AuCl_2_(κ^2^-Tpm)]Cl on Graphene

Three different ways to support the C-scorpionate Au(III) complex (prepared by ball milling or in aqueous phase) on graphene were performed: (i) wet impregnation (WI), (ii) under microwave irradiation (MW), and (iii) liquid assisted grinding (LAG). For all immobilization methods, the gold loadings on graphene of 2 wt % and 10 wt % were chosen, based on our previous experience of anchoring [AuCl_2_(κ^2^-Tpm)]Cl at other carbon materials [9].

#### 2.2.1. Wet Impregnation (WI)

To 20 mL of distilled water, graphene and [AuCl_2_(κ^2^-Tpm)]Cl were added, at room temperature, under vigorous stirring for 24 h. The solid particles were then separated from the liquid phase and dried at 50 °C overnight. The heterogenization process was monitored by ultraviolet-visible spectroscopy (UV-vis) analysis of aliquots of the liquid phase media taken during the heterogenization process (and always returned to the solution).

#### 2.2.2. Microwave Irradiation (MW)

Graphene and [AuCl_2_(Tpm)]Cl (prepared by mechanochemical synthesis or in liquid phase), in the required amounts to attain the desired gold loadings (2 wt % and 10 wt %), were added to distilled water (10–15 mL) in a 30 mL Pyrex tube and subjected to microwave irradiation (25 W) at 40 °C and 650 rpm for 30 min. The heterogenization process was then evaluated by UV-vis analysis of an aliquot of the liquid phase medium. Both phases were separated, with the solid particles collected and dried overnight at 40 °C.

#### 2.2.3. Liquid Assisted Grinding (LAG)

The Emax High Energy Ball Mill Retsch^®^ equipment was used. The parameters and conditions used were: 3 stainless steel milling balls, 15 min, and 800 rpm. After the reaction time, the grinding jar, initially containing a mixture of graphene [AuCl_2_(κ^2^-Tpm)]Cl (prepared by mechanochemical synthesis or in liquid phase) and distilled water (1–2 mL), was dried overnight at 50 °C and the solid was collected. The possibility of obtaining graphene-anchored [AuCl_2_(κ^2^-Tpm)]Cl by its in situ synthesis (i.e., in the presence of graphene) was tested under several reaction parameters, but failed to produce the expected results.

### 2.3. Microwave-Assisted Oxidation Reactions

The microwave-assisted oxidation reactions were performed in the presence of the above prepared materials as catalysts: [AuCl_2_(κ^2^-Tpm)]Cl prepared by mechanochemical synthesis ([AuCl_2_(κ^2^-Tpm)]Cl_BM) and in liquid phase ([AuCl_2_(κ^2^-Tpm)]Cl_LP), and graphene heterogenized [AuCl_2_(κ^2^-Tpm)]Cl by WI, MW, and LAG. For graphene-supported materials, both samples with gold loadings of 2 wt % and 10 wt % were used.

For glycerol oxidation reactions, 5 mmol of substrate, the desired amount of catalyst to attain a 0.2–0.4 %mol ratio of catalyst relative to substrate, and 10 mmol of *tert*-butyl hydroperoxide TBHP (70% aq. sol.) were added to a 10 mL capacity cylindrical Pyrex tube. The focused microwave irradiation (25 W) occurred for 2 h at the desired temperature (50 °C or 80 °C).

For HMF and HFCA substrates, 5 mmol were mixed with the catalyst (in a 0.2–0.4 %mol ratio of catalyst vs. HMF or HFCA) and 10 mmol of TBHP 70%. The MW irradiation (25 W) was performed for 2 h at 50 °C or 80 °C.

Blank experiments were performed for all the microwave-assisted oxidation reactions, in gold-free systems but in the presence of graphene (where applicable), and in Au and graphene-free medium.

The reactions products were analyzed by ^1^H NMR using deuterated acetone as a solvent.

The heterogenized catalysts were recovered from the reaction media and the recycling experiments conducted consecutively under the found optimized conditions using fresh reagents.

## 3. Results

### 3.1. Dry Mechanochemical and Liquid Phase Synthesis of [AuCl_2_(Tpm)]Cl

With the aim of improving the greenness of the C-scorpionate hydrotris(1*H*-pyrazol-1-yl)methane dichloro-gold(III) complex [AuCl_2_(κ^2^-Tpm)]Cl (Tpm = HCpz_3_; pz = pyrazol-1-yl, Scheme 3a) synthetic procedure [9] by avoiding the use of precious water as a solvent and washing agent, herein [AuCl_2_(κ^2^-Tpm)]Cl was synthesized by dry mechanochemistry (Scheme 3b) as the first C-scorpionate being formed by mechanochemical synthesis.

Being aware that the mechanochemical route is controlled by various factors (e.g., milling jars and balls, rotation speed, or milling time) that should be optimized to obtain the desired product, firstly, the Planetary Emax High Energy Ball Mill with two stainless steel milling jars and stainless steel milling balls was used. Several conditions, such as ball-to-powder ratio (1.2:1 to 8:1), rotation frequency (100–800 min^−1^), and milling time (5–30 min), were tested. The optimum conditions, leading to the highest yield of [AuCl_2_(κ^2^-Tpm)]Cl (89.9%), were found to be a ball-to-powder ratio of 4:1, rotating at 500 rpm during 15 min. The resulting bright yellow solid was vacuum-dried overnight at 60 °C and characterized by FTIR and ^1^H NMR spectroscopy. The spectroscopic properties of the mechanochemically prepared compound fully match those reported [9] for [AuCl_2_(κ^2^-Tpm)]Cl, indicating the authenticity of the newly obtained compound. In fact, its IR spectra exhibit the typical ν(C=C) and ν(C=N) bands of coordinated pyrazolyl rings (1635.64 br and 1527.62 cm^−1^), as well as strong intensity ν(Au–Cl) bands: 357.67 cm^−1^ [ν(Au-Cl)] asymmetric and 300.13 [ν(Au-Cl)] symmetric. In addition, the ^1^H NMR spectrum (run at −47 °C to avoid the known room temperature pyrazolyl groups fluxional behavior [9] in CD_3_OD) displays a two-line pattern (2:1) for the protons of the pyrazolyl rings, in accordance with the κ^2^-coordination of the Tpm ligand (see Figure 1).

**Figure 2:**
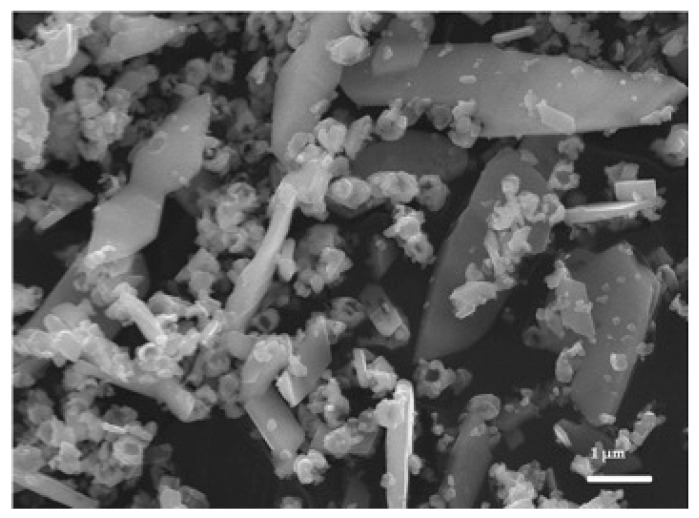
SEM micrograph of the hydrotris(1*H*-pyrazol-1-yl)methane dichloro-gold(III) complex [AuCl_2_(κ^2^-Tpm)]Cl (Tpm = HCpz_3_; pz = pyrazol-1-yl) synthesized by mechanochemistry using the Planetary Emax High Energy Ball Mill.

With the knowledge that the critical issue with the dry mechanochemical route is the possibility of product contamination (due to the abrasion of milling balls and jars), the morphology of the mechanochemically prepared [AuCl_2_(κ^2^-Tpm)]Cl was investigated by a scanning electron microscope (SEM) equipped with energy dispersive X-ray spectroscopy (EDS), depicted in Figure 2. EDS shows the relative weight concentration on the surface of the mechanochemical product. It confirms the presence of C, N, Cl, and Au on the compound. No contamination from the milling reactor was found (see Figure S1a, ESI).

Besides being a solvent-free method, this mechanochemical synthesis by neat grinding resulted in a significantly higher yield of [AuCl_2_(κ^2^-Tpm)]Cl (89.9%) than the reported (72.0% yield) [9] for the traditional method which uses water as a solvent.

**Figure 3:**
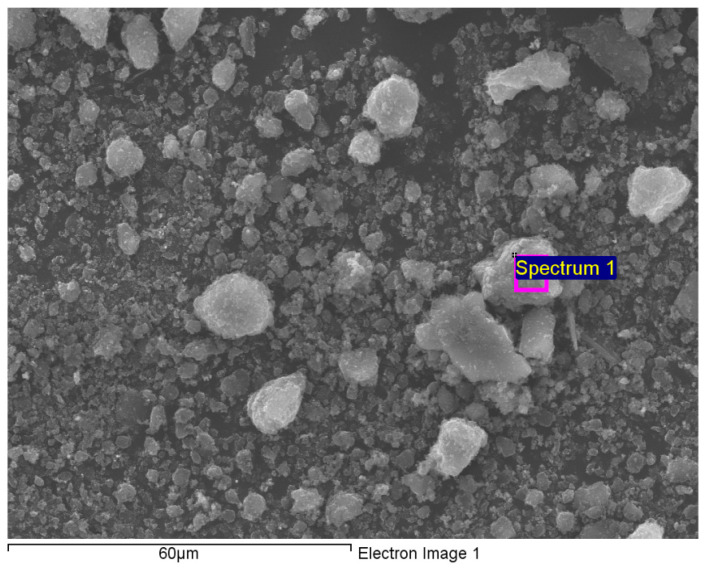
SEM micrograph of the mechanochemical product from reaction of an equimolar amount of HAuCl_4_·3H_2_O and Tpm, using the Planetary Ball Mill PM 100.

The mechanochemical synthesis of the C-scorpionate Au(III) complex was then performed with the same amount of reactants at the Planetary Ball Mill PM 100 under the previous optimized conditions: ball-to-powder ratio of 4:1, rotating at 500 rpm during 15 min. Surprisingly, a black solid was formed and this was vacuum-dried overnight at 60 °C and analyzed by SEM/EDS (Figure 3). The nomenclature used to denote this black solid is AuClTpm_PM.

The PM 100 ball milled sample (Figure 3) exhibits the same type of surface but larger pieces and is more scattered than the presented [AuCl_2_(κ^2^-Tpm)]Cl synthesized in the Emax High Energy Ball Mill ([AuCl_2_(κ^2^-Tpm)]Cl_BM, Figure 2). Moreover, EDS confirmed the presence of iron and chromium (steel constituents) at the surface of the material (Figure S1b, ESI), indicating that contamination occurred.

**Figure 4:**
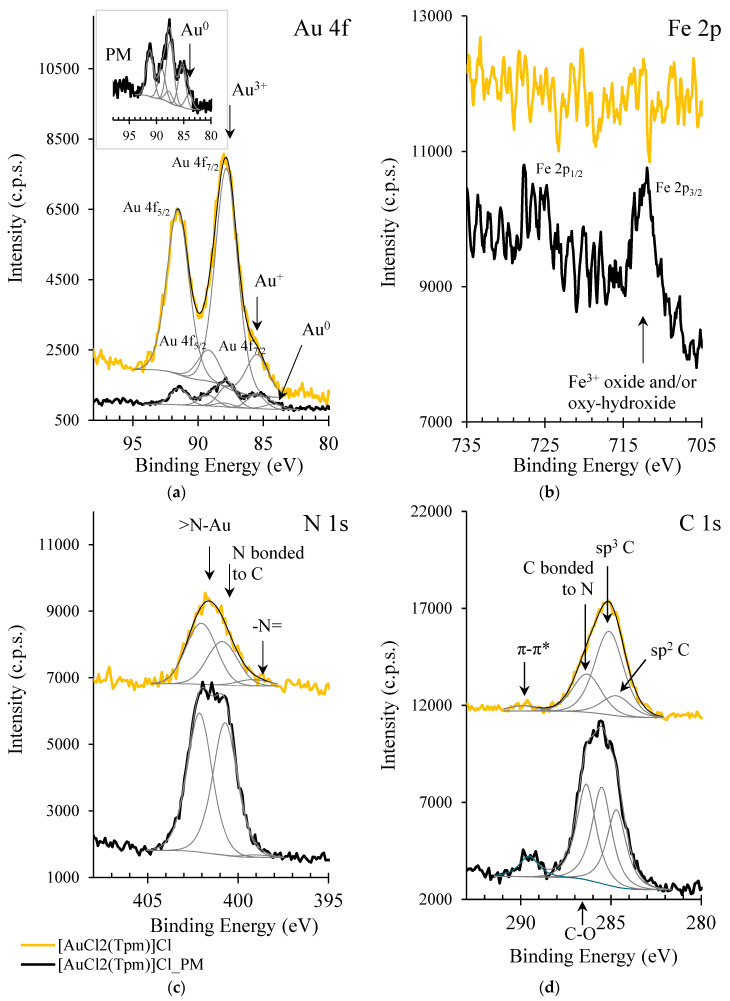
XPS regions of [AuCl_2_(κ^2^-Tpm)]Cl (yellow spectrum) and AuClTpm_PM (black spectrum): (**a**) Au 4f, (**b**) Fe 2p, (**c**) N 1s, and (**d**) C 1s.

XPS analysis was performed for the products obtained in both ball mills. It shows that the C-scorpionate gold(III) complex prepared in the Emax High Energy Ball Mill, [AuCl_2_(κ^2^-Tpm)]Cl_BM, has a composition closer to the predicted one than the sample prepared in the PM 100 Ball Mill. However, the atomic ratio N/Au = 3.4:1 shows that [AuCl_2_(κ^2^-Tpm)]Cl_BM has more gold atoms per C-scorpionate unit (~2 atoms of Au/“Tpm”) than that anticipated by the proposed chemical structure (Figure 1 and Table S1). In addition, some oxygen is detected (7.0%). In the PM ball milled sample, gold is barely detected. This effect may be due to an aggregation of gold atoms which, in XPS, can result in an underestimation of the relative amount of gold existing in the sample, since part of the photoelectron signal coming from gold atoms located inside an atomic cluster or a nanoparticle is attenuated by the outermost layers. The XPS signal of chemical species that are not aggregated and therefore more exposed to the XPS detector are not as attenuated. In fact, analyzing the detailed XPS regions (Figure 4), Au 4f region shows very clear differences: [AuCl_2_(κ^2^-Tpm)]Cl_BM, which is a yellow powder, is composed of two doublets, with spin orbit split of 3.7 eV, with Au 4f_7/2_ components centered at 87.4 ± 0.1 eV (the main doublet) and 85.1 ± 0.1 eV (with lower relative intensity). These components are assigned to Au^3+^ and Au^+^, respectively. It is worth noting that these two oxidation states co-exist in the HAuCl_4_·3H_2_O precursor used for the synthesis of the complex [29]. AuClTpm_PM, which is a black powder, presents an Au 4f region with a third doublet, with the Au 4f_7/2_ component centered at 84.0 ± 0.1 eV attributed to Au^0^ [29]. The other two doublets are at the same positions as those of [AuCl_2_(κ^2^-Tpm)]Cl_BM, but with different proportions. Both the quantitative and qualitative analyses seem to show that the PM ball milling reduces gold-generating Au nanoparticles. Moreover, iron was also detected in the form of iron oxide and/or oxy-hydroxide in AuClTpm_PM (Figure 4), with the only source of iron being the ball mill jar and/or the balls used, made of stainless steel 316 (iron is absent in [AuCl_2_(κ^2^-Tpm)]Cl formed in the Emax High Energy Ball Mill). XPS, being a less in-depth technique, did not detect the Cr contamination observed by SEM/EDS analysis.

Regarding the Tpm ligand, N 1s of [AuCl_2_(κ^2^-Tpm)]Cl_BM was fitted with three peaks, centered at 399.2 ± 0.1 eV, 400.9 ± 0.1 eV, and 402.0 ± 0.1 eV, assigned to pyridinic nitrogen, nitrogen bonded to carbon (typical of pyrazole groups), and nitrogen with an electron donor character when coordinating with gold, respectively. AuClTpm_PM also presents the two main peaks, centered at 400.7 ± 0.1 eV and 402.1 ± 0.1 eV, and an almost imperceptible peak at 399.0 eV. C 1s was fitted with the two peaks predicted for the Tpm: one assigned to aromatic carbon atoms (centered at 284.7 ± 0.1 eV, which was used as BE reference for charge shift correction) and another from carbon bonded to nitrogen (centered at 286.3 ± 0.1 eV) [30]. As expected, the area (C-N) is approximately twice the area (Csp^2^). An additional peak was needed to fit the C 1s profile: the main peak centered at 285.1 ± 0.1 eV, which is attributed to aliphatic carbon atoms. At higher BE, close to 290 eV, a small peak from π-π* excitation energy losses is detected [30].

In terms of performance, planetary ball mills are known for their mixing and size-reduction processes. A PM100 reactor can provide the energy input for a mechanical and chemical transformation due to the centrifugal forces present. The major drawback is the shape of a reactor that is not suited for very high-energy milling (more common for synthetic chemistry and metal alloying). The Emax High Energy Ball Mill is an entirely new type of mill for high-energy input. The unique combination of high friction and impact results in extremely fine particles (suitable for synthetic purposes) within the shortest amount of time (preventing corrosion by pitting of the reactors and contamination of the samples). The extremely high centrifugal forces of a planetary ball mill result in very high pulverization energy and therefore short grinding times. Another factor to consider is the energy input due to the number of milling balls. A balance is required to obtain a mechanochemical reaction and not only a reduction of size (mechanical procedure). This is of vital importance to obtain new samples with defined oxidation states.

Thus, ball mill equipment, milling jars, and balls should be chosen according to the type of reaction and operation conditions, due to the impact of the milling balls on the inner walls of the jars and the materials to be milled. In addition, the compatibility of materials should be considered. In this respect, auric acid deserves the greatest attention as a reagent.

### 3.2. Heterogenization of [AuCl_2_(κ^2^-Tpm)]Cl on Graphene (G)

**Figure 5:**
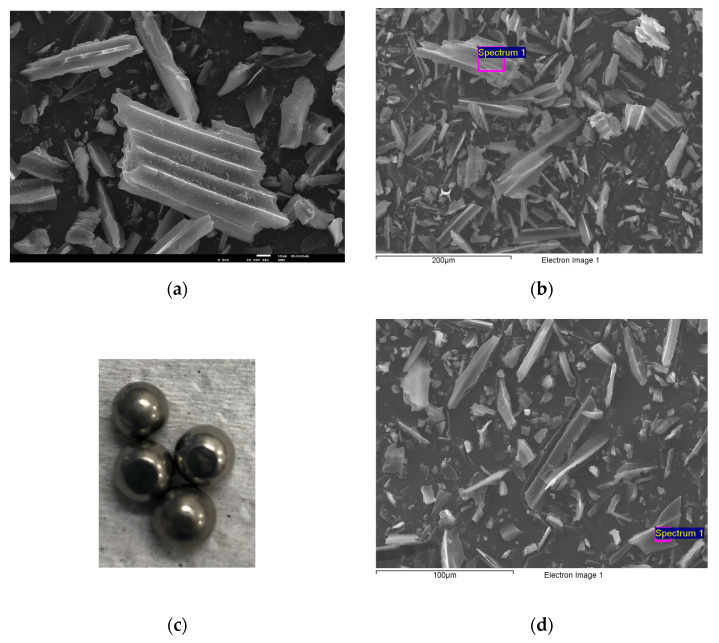
SEM micrographs of pristine graphene sheets (**a**) before and after (**b**) mechanochemical treatment with the Planetary Ball Mill PM 100. The image in (**c**) shows milling balls flated due to collisions, while (**d**) shows mechanochemical treatment after collisions in the Emax High Energy Ball Mill.

Before being used as heterogenization support, commercial graphene (Graphene, Sigma-Aldrich) was characterized by SEM/EDS (Figure 5a and Figure S1c), FTIR-ATR, and powder X-ray diffraction (PXRD) displayed in Figures S2 and S3, respectively (ESI). All characterizations are in accord with the expected patterns [31,32].

To try to understand if the contamination would also affect pristine graphene, a milling test under the planned immobilization conditions (see experimental) was performed, followed by SEM/EDS analysis (Figure 5b for PM 100 ball mill and 5d for the Emax ball mill). Contamination by iron and chromium was clearly detected for graphene that had undergone mechanochemical treatment with the Planetary Ball Mill PM 100 (Figure S1d, ESI). Moreover, the original form of the stainless-steel milling balls was lost within the experiment, as depicted in Figure 5c. Therefore, the anchorage of [AuCl_2_(κ^2^-Tpm)]Cl by mechanochemical treatment was performed only with the Emax High Energy Ball Mill, where the contamination was considered negligible.

The heterogenization of the C-scorpionate gold(III) complex [AuCl_2_(κ^2^-Tpm)]Cl, either prepared by neat grinding or in liquid phase, into graphene (G) was performed by three different methods: (i) wet impregnation, (ii) microwave irradiation, and (iii) liquid assisted grinding (see experimental). Liquid assisted grinding was chosen for the immobilization in view of its advantages since it is as an extension of traditional solvent-free mechanochemical techniques by which a small amount of liquid is used as an additive to enhance and/or control reactivity, relative to neat grinding. In fact, it has been successfully applied in the screening of inclusion compounds, cocrystals, salts, solvates, and polymorphs. Another advantage is that the liquid disperses the energy produced by the friction in a more homogeneous process, making it a more efficient process for synthetic chemistry [33].

The mechanochemical methodology was the most effective in the incorporation of Au amounts close to the theoretical loadings (2 wt % and 10 wt %), as presented in Table 1, followed by microwave-assisted impregnation. In addition, the intake of gold detected in the material obtained by immobilization of [AuCl_2_(κ^2^-Tpm)]Cl prepared by mechanochemistry ([AuCl_2_(κ^2^-Tpm)]Cl_BM@G_LAG) was always higher than that attained for the gold(III) complex prepared in liquid phase ([AuCl_2_(κ^2^-Tpm)]Cl_LP@G_LAG).

**Figure 6:**
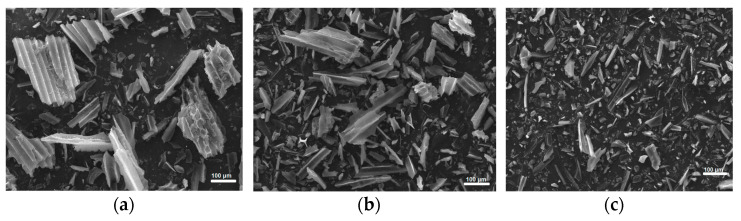
SEM micrographs of [AuCl_2_(κ^2^-Tpm)]Cl_BM supported on graphene (G) by (**a**) WI ([AuCl_2_(κ^2^-Tpm)]Cl_BM@G_WI, (**b**) MW ([AuCl_2_(κ^2^-Tpm)]Cl_BM@G_MW, and (**c**) LAG ([AuCl_2_(κ^2^-Tpm)]Cl_BM@G_LAG).

The structure and morphology of the graphene-supported gold(III) C-scorpionate complex by the above techniques were characterized by SEM and EDS. Figure 6 depicts the micrographs obtained with [AuCl_2_(κ^2^-Tpm)]Cl prepared by mechanochemistry ([AuCl_2_(κ^2^-Tpm)]Cl_BM).

All tested techniques were shown to efficiently produce graphene-supported [AuCl_2_(κ^2^-Tpm)]Cl, without losing the structure of the support. It is also clear from Figure 6 that LAG affords smaller-size particles compared with the previous WI or MW techniques.

### 3.3. Microwave-Assisted Oxidation Reaction of Glycerol to DHA

**Scheme 4:**
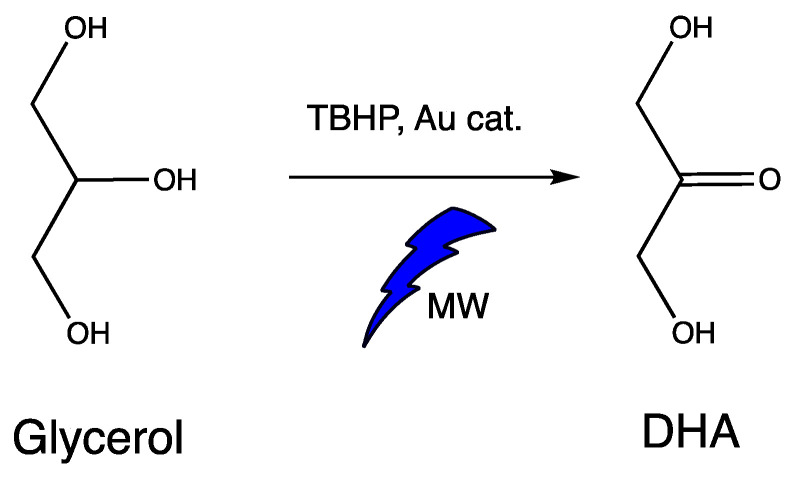
Microwave-assisted oxidation of glycerol to dihydroxyacetone (DHA).

The solvent-free oxidation of glycerol to dihydroxyacetone (DHA, Scheme 4) using [AuCl_2_(κ^2^-Tpm)]Cl (synthesized by the two different methods, see Section 3.1) as a catalyst, in homogeneous and heterogeneous conditions, was tested under microwave irradiation. It allowed, for the first time, the evaluation of the catalytic performance of these new materials in terms of alcohol conversion, product yield, turnover number (TON, number of moles of product per number of moles of catalyst), or turnover frequency (TOF, h^−1^, TON/reaction time).

The catalytic experiments were initiated by choosing an aqueous solution of *tert*-butyl hydroperoxide (THBP) as an oxidizing agent and using a low amount of catalyst (10–20 μmol) in solvent-free conditions. THBP was preferred to hydrogen peroxide, for example, because of its lower handling risk. The oxidation of glycerol under the above-mentioned conditions led to dihydroxyacetone as the main product. As shown in Table 2 and Table 3 (selected results), a significant selectivity for the oxidation of the secondary OH group of glycerol was found for this gold(III) catalyst at low catalyst load and temperature. In homogeneous conditions, such DHA selectivity was maintained for higher amounts of catalyst but not for higher temperatures, while for the reaction catalyzed by the heterogenized catalyst, both increases in catalyst amount and reaction temperature led to a drastic reduction in DHA selectivity, indicating the formation of by-products (hydroxypyruvic acid was detected in both homo- and heterogeneous catalysis).

Both complexes, prepared in conventional liquid phase according to reference [9] and by dry ball milling, exhibited similar catalytic activity under homogeneous conditions, leading to yields of DHA up to 74% (Table 2, entry 5). On the contrary, the parent compound, HAuCl_4_·3H_2_O, presented a much lower catalytic ability to convert glycerol into dihydroxyacetone (Table 2, entries 7–9).

Heterogenized catalysts clearly show an improved catalytic performance relative to that presented when used neat (compare Table 2 and Table 3). In fact, under optimized conditions (n(TBHP)/n(glycerol) = 2, 20 μmol Au, 25 W microwave irradiation at 650 rpm for 2 h), [AuCl_2_(Tpm)]Cl, either prepared in conventional liquid phase according to reference [9] or by dry ball milling, attained up to 87% yield of DHA (entries 1 and 4 of Table 3), whereas a maximum of 74% yield of DHA was reached under homogeneous conditions (Table 2, entry 5). Moreover, when comparing the supported complexes in graphene by the three heterogenization procedures (WI, MW, and LAG), in general, the best results in terms of activity and selectivity were obtained for the materials resulting from the immobilization of [AuCl_2_(Tpm)]Cl_BM (or [AuCl_2_(Tpm)]Cl_LP) by liquid assisted grinding (Table 3, entries 1–6). In addition, it was found that a lower loading of Au catalyst at the graphene support considerably favors the desired DHA formation (e.g., compare entries 1 and 3 of Table 3). Indeed, further apparent oxidation of DHA to hydroxypyruvic acid was detected by NMR spectroscopy for the catalysts presenting the highest amount of gold (9.72 wt %) immobilized at graphene. The formation of hydroxypyruvic acid from DHA foresees the oxidation of a terminal OH group while preserving the other terminal OH group. An alternative formation of hydroxypyruvic acid could result via formation of glyceraldehyde (by oxidation of one terminal OH group of glycerol) and its further oxidation to glyceric acid. However, neither glyceraldehyde nor glyceric acid were detected in the present work.

**Figure 7:**
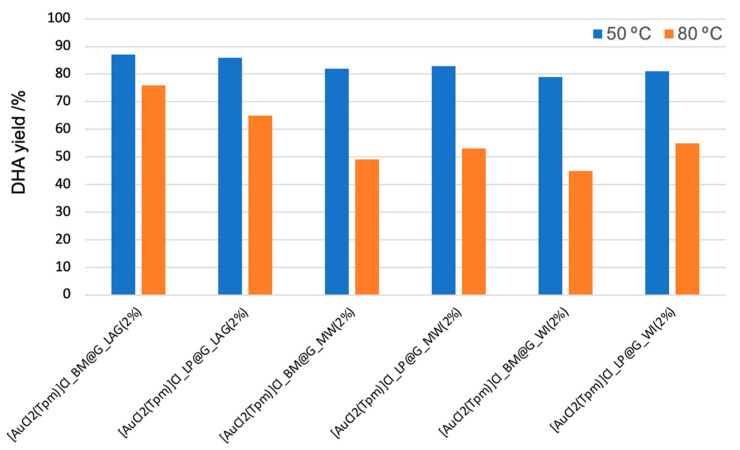
DHA yields achieved by the oxidation of glycerol at temperatures of 50 °C and 80 °C, catalyzed by [AuCl_2_(Tpm)]Cl (synthesized in liquid phase or by dry grinding) and supported at graphene by different methods (wet impregnation, microwave irradiation, and liquid assisted grinding).

The successful oxidation of glycerol to DHA is significantly dependent on the reaction temperature, despite the heterogenization method. Although an increase in the MW temperature from 50 to 80 °C led to higher glycerol conversions (Table 3), a decrease in DHA yields relative to that attained at 50 °C was always observed (Figure 7). The formation of hydroxypyruvic acid was also detected in the reaction mixtures at 80 °C.

**Figure 8:**
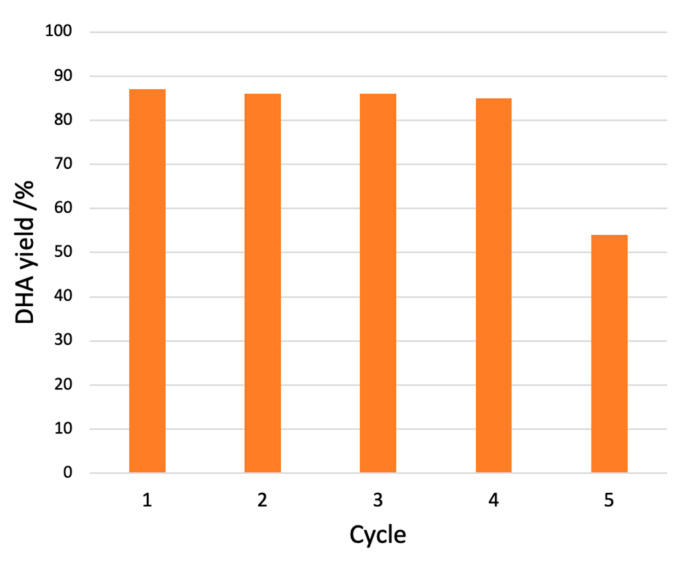
Recycling assays (in optimized conditions) using [AuCl_2_(Tpm)]Cl_BM@G_LAG as a catalyst.

[AuCl_2_(Tpm)]Cl_BM@G_LAG, which exhibited the best catalytic performance (Table 3, entry 1), was chosen for the recycling experiments. Its stability was evaluated by recovering it and performing consecutive catalytic oxidation cycles with fresh reagents. Catalyst [AuCl_2_(Tpm)]Cl_BM@G_LAG, besides being synthesized and heterogenized by green procedures, has displayed the additional advantage of being easily recovered and reused in up to four cycles without significant loss of activity while maintaining its selectivity towards the formation of DHA (Figure 8).

### 3.4. Microwave-Assisted Oxidation Reaction of HMF to DFF

Current base-free (greener and less expensive) catalytic systems for HMF oxidation suffer from low yield and selectivity [6]. As such, efficient catalysts focusing on base-free conversion systems are required.

**Scheme 5:**
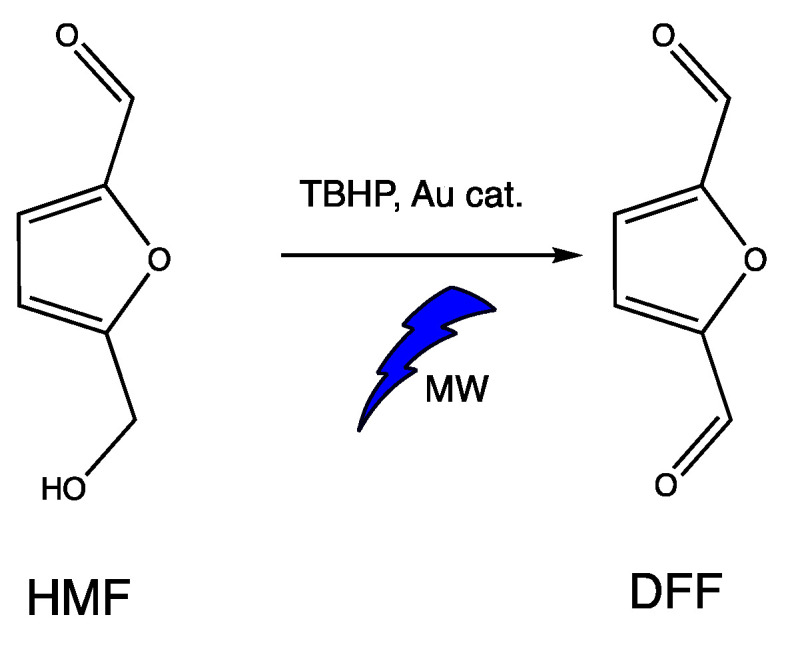
Microwave-assisted oxidation of 5-(hydroxymethyl)furfural (HMF) to furan-2,5-dicarbaldehyde (DFF).

Herein, complex [AuCl_2_(κ^2^-Tpm)]Cl (synthesized in liquid phase and by dry grinding, see Section 3.1) was used as a catalyst for the microwave-assisted, solvent-free oxidation of 5-(hydroxymethyl)furfural (HMF) to furan-2,5-dicarbaldehyde (DFF, Scheme 5) by *tert*-butyl hydroperoxide (70% aq. sol.) in homogeneous and heterogeneous conditions. This allowed,, for the first time, the evaluation of the catalytic performance of these new materials in terms of substrate conversion, product yield, and turnover frequency (Table 4). In such a base-free medium, the oxidation of HMF is expected to proceed via the DFF route (Route B, Scheme 2) [6] due to a faster alcohol group oxidation than aldehyde group oxidation in HMF.

Our gold catalysts displayed encouraging efficiency for HMF oxidation. In fact, all the Au-based catalysts, despite the synthetic or immobilization procedures used, are remarkably selective to DFF formation (Table 4), as determined by ^1^H NMR spectroscopy.

As observed previously for the glycerol oxidation, heterogenized catalysts clearly show an improved catalytic performance relative to that presented when used neat (e.g., compare entries 1 and 2 of Table 4). In fact, under optimized conditions (n(TBHP)/n(HMF) = 2, 20 μmol Au, 25 W microwave irradiation at 650 rpm for 2 h), [AuCl_2_(Tpm)]Cl, either prepared in conventional liquid phase according to reference [9] or by dry ball milling, attained up to 84% yield of DFF (entries 4 and 8 of Table 3), whereas a maximum of 64% yield of DFF was reached under homogeneous conditions (entries 1 and 6 of Table 4).

The best DFF yield was achieved in the presence of the hybrid catalyst, formed by mechanochemistry and supported by the microwave irradiation procedure, [AuCl_2_(Tpm)]Cl_BM@G_MW (84%, entry 4 of Table 4). It is known that the efficiency of heterogenized gold catalysts is strongly influenced by supporting materials, with graphene appearing to be remarkably suitable regarding selectivity to DFF.

A recycling assay using [AuCl_2_(Tpm)]Cl_BM@G_MW as a catalyst was performed by recovering it from the reaction medium and performing consecutive catalytic oxidation cycles with fresh reagents. However, only 45% of its initial catalytic activity was preserved after the first reuse run, indicating the occurrence of leaching during the catalytic reaction or the catalyst recovery process.

### 3.5. Microwave-Assisted Oxidation Reaction of HFCA to FFCA

**Scheme 6:**
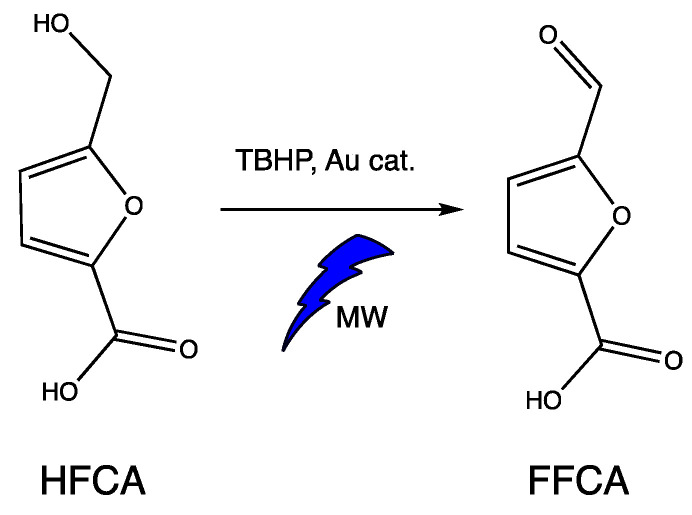
Microwave-assisted oxidation of 5-hydroxymethyl-2-furancarboxylic acid (HFCA) oxidation to 5-formylfuran-2-carboxylic acid (FFCA).

The ability of our gold catalysts to assist the conversion of the product from alkaline oxidation of HMF, 5-hydroxymethyl-2-furancarboxylic acid (HFCA, Route A, Scheme 2) was also investigated. Thus, complex [AuCl_2_(κ^2^-Tpm)]Cl synthesized by dry grinding (see Section 3.1) was used as catalyst towards the microwave-assisted, solvent-free oxidation of HFCA to 5-formylfuran-2-carboxylic acid (FFCA, Scheme 6) by *tert*-butyl hydroperoxide (70% aq. sol.) in homogeneous and heterogeneous conditions. Selected results are displayed in Table 5.

Complex [AuCl_2_(Tpm)]Cl_BM, used either neat or immobilized at graphene, exhibited a much lower activity towards the oxidation of HFCA than that achieved for the oxidation of glycerol or HMF under the same reaction conditions (50 °C, see Table 5). A maximum of 58% yield of FFCA was achieved when the temperature was raised to 80 °C.

In addition, recycling tests of [AuCl_2_(Tpm)]Cl_BM@G_LAG(2%) were performed as before (see Section 3.3 and Section 3.4), but the catalyst appeared to suffer from deactivation and degradation problems, showing a much-reduced catalytic activity in successive cycles.

## 4. Conclusions

In this work, a simple and sustainable route was (for the first time) successfully developed to prepare a mechanochemical synthesized C-scorpionate Au(III) complex with the absence of harsh chemicals and in a cost-effective way. This dry mechanochemical route offers the potential to significantly decrease the environmental footprint (i.e., energy, chemical usage, and disposal). Mechanochemical treatment was also used, along with microwave irradiation and wet impregnation procedures, to immobilize the C-scorpionate Au(III) complex at graphene. The prepared hybrid catalysts were revealed to be more promising than neat Au(III) complex for the tested microwave-assisted oxidation reactions, leading to higher yields and selectivities for the desired products and allowing an easy separation and recycling.

The success of the herein disclosed results deserves further development in designing efficient catalysts and, combined with molecular simulation, may provide new insights to guide the process of achieving an optimized process to convert such renewable and sustainable feedstocks into chemical intermediates and polymeric materials.

## Figures and Tables

**Table 1:** Gold loadings obtained by ICP-AES after heterogenization of graphene by different methods.

[AuCl_2_(κ^2^-Tpm)]Cl	Impregnation Method	Au (wt %.) (2% Max.)	Au (wt %.) (10% Max.)
mechanochemistry ([AuCl_2_(κ^2^-Tpm)]Cl_BM)	WI	1.18	6.56
	MW	1.61	8.45
	LAG	1.97	9.72
liquid phase ([AuCl_2_(κ^2^-Tpm)]Cl_LP)	WI	1.25	5.84
	MW	1.16	9.18
	LAG	1.89	9.22

**Table 2:** Selected results ^a^ for the MW-assisted oxidation of glycerol to dihydroxyacetone in homogenous conditions.

Entry	T (°C)	Catalyst ^b^	Catalyst (μmol)	DHA Yield ^c^ (%)	Conversion (%)	TOF ^d^ (h^−1^)
1	50		10	25	26	63
2	50	[AuCl_2_(Tpm)]Cl_LP	20	73	74	91
3	80		10	10	80	25
4	50		10	23	23	58
5	50	[AuCl_2_(Tpm)]Cl_BM	20	74	76	93
6	80		10	10	84	25
7	50		10	2	30	5
8	50	HAuCl_4_·3H_2_O	20	16	93	20
9	80		10	8	100	20
10	50	-	-	0	1	-

^a^ Reaction conditions: 5 mmol of substrate; 10 mmol of TBHP (70% aq. sol.); 650 rpm; 25 W; 2 h reaction time. ^b^ LP—prepared in liquid phase [9]; BM—prepared by dry ball milling. ^c^ Yield (%) = moles of DHA/100 moles of glycerol. ^d^ TOF = (moles of DHA/mole of catalyst)/time.

**Table 3:** Selected results ^a^ for the MW-assisted oxidation of glycerol in heterogeneous conditions.

Entry	T (°C)	Catalyst	DHA Yield ^b^ (%)	Conversion (%)	TOF ^c^ (h^−1^)
1	50	[AuCl_2_(Tpm)]Cl_BM@G_LAG(2%)	87	93	109
2	80	[AuCl_2_(Tpm)]Cl_BM@G_LAG(2%)	76	96	95
3	50	[AuCl_2_(Tpm)]Cl_BM@G_LAG(10%)	51	91	64
4	50	[AuCl_2_(Tpm)]Cl_LP@G_LAG(2%)	86	90	108
5	80	[AuCl_2_(Tpm)]Cl_LP@G_LAG(2%)	65	97	82
6	50	[AuCl_2_(Tpm)]Cl_LP@G_LAG(10%)	56	97	70
7	50	[AuCl_2_(Tpm)]Cl_BM@G_MW(2%)	82	88	103
8	80	[AuCl_2_(Tpm)]Cl_BM@G_MW(2%)	49	77	61
9	50	[AuCl_2_(Tpm)]Cl_BM@G_MW(10%)	77	80	96
10	50	[AuCl_2_(Tpm)]Cl_LP@G_MW(2%)	83	87	104
11	80	[AuCl_2_(Tpm)]Cl_LP@G_MW(2%)	53	91	66
12	50	[AuCl_2_(Tpm)]Cl_LP@G_MW(10%)	47	94	59
13	50	[AuCl_2_(Tpm)]Cl_BM@G_WI(2%)	79	87	99
14	80	[AuCl_2_(Tpm)]Cl_BM@G_WI(2%)	45	68	56
15	50	[AuCl_2_(Tpm)]Cl_BM@G_WI(10%)	76	99	95
16	50	[AuCl_2_(Tpm)]Cl_LP@G_WI(2%)	81	88	101
17	80	[AuCl_2_(Tpm)]Cl_LP@G_WI(2%)	55	97	69
18	50	[AuCl_2_(Tpm)]Cl_LP@G_WI(10%)	52	89	65
19	50	Graphene (G)	1	2	-

^a^ Reaction conditions: 5 mmol of glycerol; 10 mmol of TBHP 70%; 20 μmol Au; 650 rpm; 25 W; 2 h reaction time. BM—prepared by dry milling; LP—prepared in liquid phase [9]; G—graphene; WI—wet impregnation; MW—microwave irradiation; LAG—liquid assisted grinding. ^b^ Yield (%) = moles of DHA/100 moles of glycerol. ^c^ TOF = (moles of DHA/mole of Au)/time.

**Table 4:** Selected results ^a^ for the MW-assisted oxidation of HMF to DFF in homogeneous and heterogenous conditions.

Entry	Catalyst	DFF Yield ^b^ (%)	Conversion (%)	TOF ^c^ (h^−1^)
1	[AuCl_2_(Tpm)]Cl_BM	63	63	79
2	[AuCl_2_(Tpm)]Cl_BM@G_LAG(2%)	72	72	90
4	[AuCl_2_(Tpm)]Cl_BM@G_MW(2%)	84	84	105
5	[AuCl_2_(Tpm)]Cl_BM@G_WI(2%)	74	75	93
6	[AuCl_2_(Tpm)]Cl_LP	64	65	80
7	[AuCl_2_(Tpm)]Cl_LP@G_LAG(2%)	71	71	89
8	[AuCl_2_(Tpm)]Cl_LP@G_MW(2%)	83	83	104
9	[AuCl_2_(Tpm)]Cl_LP@G_WI(2%)	72	73	90
10	Graphene (G)	2	6	3
11	-	0	1	-

^a^ Reaction conditions: 5 mmol of HMF; 10 mmol of TBHP 70%; 20 μmol Au; 650 rpm; 25 W; 2 h reaction time. BM—prepared by dry milling; LP—prepared in liquid phase [9]; G—graphene; WI—wet impregnation; MW—microwave irradiation; LAG—liquid assisted grinding. ^b^ Yield (%) = moles of DFF/100 moles of HMF. ^c^ TOF = (moles of DFF/mole of Au)/time.

**Table 5:** Selected results ^a^ for the MW-assisted oxidation of 5-hydroxymethyl-2-furancarboxylic acid (HFCA) in homogeneous and heterogenous conditions.

Entry	T (°C)	Catalyst	FFCA Yield ^b^ (%)	Conversion (%)	TOF ^c^ (h^−1^)
1	50	[AuCl_2_(Tpm)]Cl_BM	12	13	15
2	50	HAuCl_4_·3H_2_O	8	10	10
3	50	[AuCl_2_(Tpm)]Cl_BM@G_LAG(2%)	25	26	32
4	80	[AuCl_2_(Tpm)]Cl_BM@G_LAG(2%)	58	60	73
5	50	[AuCl_2_(Tpm)]Cl_BM@G_MW(2%)	24	26	30
6	50	[AuCl_2_(Tpm)]Cl_BM@G_WI(2%)	30	35	38
7	50	Graphene (G)	0	2	-
8	50	-	0	0	-

^a^ Reaction conditions: 5 mmol of HFCA; 10 mmol of TBHP 70%; 20 μmol Au; 650 rpm; 25 W; 2 h reaction time. BM—prepared by dry milling; G—graphene; WI—wet impregnation; MW—microwave irradiation; LAG—liquid assisted grinding. ^b^ Yield (%) = moles of FFCA/100 moles of HFCA. ^c^ TOF = (moles of FFCA/mole of Au)/time.

## Supplementary Materials

The following are available online at https://www.mdpi.com/article/10.3390/nano12030362/s1. Table S1: Experimental XPS atomic concentrations (%) and atomic ratios for [AuCl_2_(Tpm)]Cl_BM and AuClTpm_PM compared to the predicted ones. Figure S1: EDS spectra (YY axis: intensity) of (a) hydrotris(1*H*-pyrazol-1-yl)methane dichloro-gold(III) complex [AuCl_2_(κ^2^-Tpm)]Cl (Tpm = HCpz_3_; pz = pyrazol-1-yl) synthesized by mechanochemistry using the planetary Emax High Energy Ball Mill; (b) the mechanochemical product from reaction of an equimolar amount of HAuCl_4_·3H_2_O and Tpm, using the planetary Ball Mill PM 100; pristine graphene sheets before (c) and (d) after mechanochemical treatment at Planetary Ball Mill PM 100 and (e) at Emax High Energy Ball Mill. Figure S2: ATR-FTIR spectrum of the graphene used for the immobilization of [AuCl_2_(Tpm)]Cl_BM. Figure S3: PXRD of the graphene used for the immobilization of [AuCl_2_(Tpm)]Cl_BM.
