# Supplementary material for: Unprecedented Mechanochemical Synthesis and Heterogenization of a C-Scorpionate Au(III) Catalyst for Microwave-Assisted Biomass Valorization
Source: Nanomaterials (Basel). 2022 Jan 23;12(3):362. doi: 10.3390/nano12030362 (PMC8839393; doi:10.3390/nano12030362)
Supplement: Supplementary file 1 [file nanomaterials-12-00362-s001.zip › nanomaterials-1557055-supplementary.pdf]

Supporting Information

for

Unprecedented Mechanochemical Synthesis and Heterogenization of a C-Scorpionate Au(III) Catalyst for Microwave-Assisted Biomass Valorization

Inês A. S. Matias [1], Pablo G. Selfa [1], Ana M. Ferraria [2,3], Ana M. Botelho do Rego [2,3], Maximilian N. Kopylovich [1], Ana P. C. Ribeiro [1,*] and Luísa M. D. R. S. Martins [1,*]

1 Centro de Química Estrutural, Institute of Molecular Sciences, and Departamento de Engenharia Química, Instituto Superior Técnico, Universidade de Lisboa, 1049-001 Lisboa, Portugal; ines.matias@tecnico.ulisboa.pt (I.A.S.M.); pablogselfa@tecnico.ulisboa.pt (P.G.S.); maximilian.kopylovich@tecnico.ulisboa.pt (M.N.K.)

2 iBB—Institute for Bioengineering and Biosciences and Departamento de Engenharia Química,
Instituto Superior Técnico, Universidade de Lisboa, Av. Rovisco Pais, 1049-001 Lisboa, Portugal; ana.ferraria@tecnico.ulisboa.pt (A.M.F.); amrego@tecnico.ulisboa.pt (A.M.B.d.R.)

3 Associate Laboratory i4HB, Institute for Health and Bioeconomy at Instituto Superior Técnico, Universidade de Lisboa, Av. Rovisco Pais, 1049-001 Lisboa, Portugal

* Correspondence: apribeiro@tecnico.ulisboa.pt (A.P.C.R.); luisammartins@tecnico.ulisboa.pt (L.M.D.R.S.M.); Tel.: +351-218-419-389 (L.M.D.R.S.M.)

Table S1 shows the XPS atomic concentrations (%) of the detected elements and relevant atomic ratios for the samples [AuCl$_2$(Tpm)]Cl_BM and AuClTpm_PM.

Table S1. – Experimental XPS atomic concentrations (%) and atomic ratios for [AuCl$_2$(Tpm)]Cl_BM and AuClTpm_PM compared to the predicted ones.

	[AuCl$_2$(Tpm)]Cl	AuClTpm_PM	Predicted
	Atomic Concentrations (%)		
Au	5.0	0.5	5
Cl	11.9	8.0	15
C	59.6	63.6	50
O	7.0	2.1	–
N	16.7	25.9	30
	Atomic ratios		
Cl/Au	2.4	17.7	3
N/Au	3.4	57.7	6

Figure S1 shows the EDS spectra (YY axis: intensity) of the different materials obtained by using the planetary Emax High Energy Ball Mill and the Planetary Ball Mill PM 100.

(a)

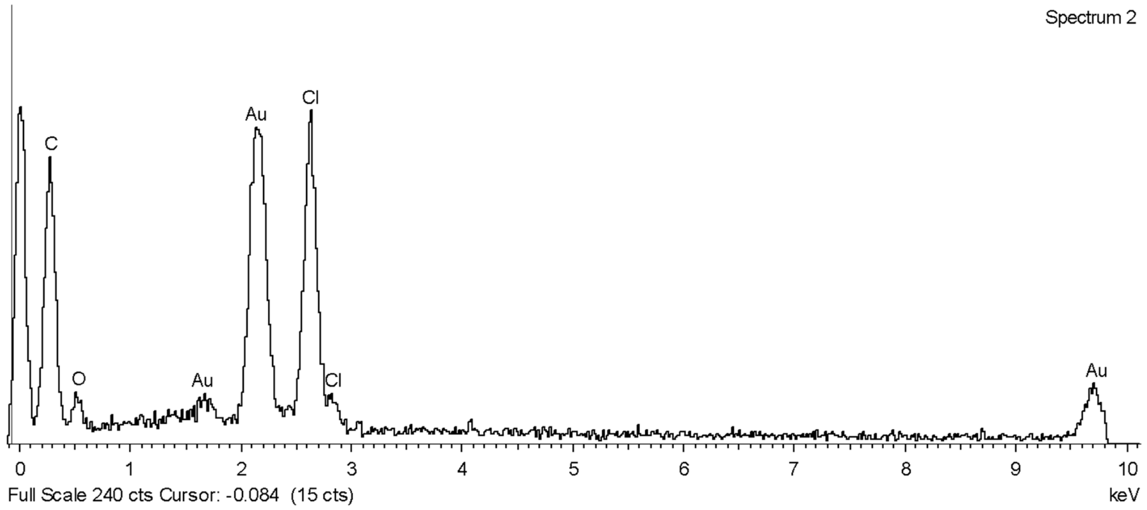
Spectrum 2
C
O
Au
Au
Cl
Cl
Au
0 1 2 3 4 5 6 7 8 9 10
Full Scale 240 cts Cursor: -0.084 (15 cts)
keV

(b)

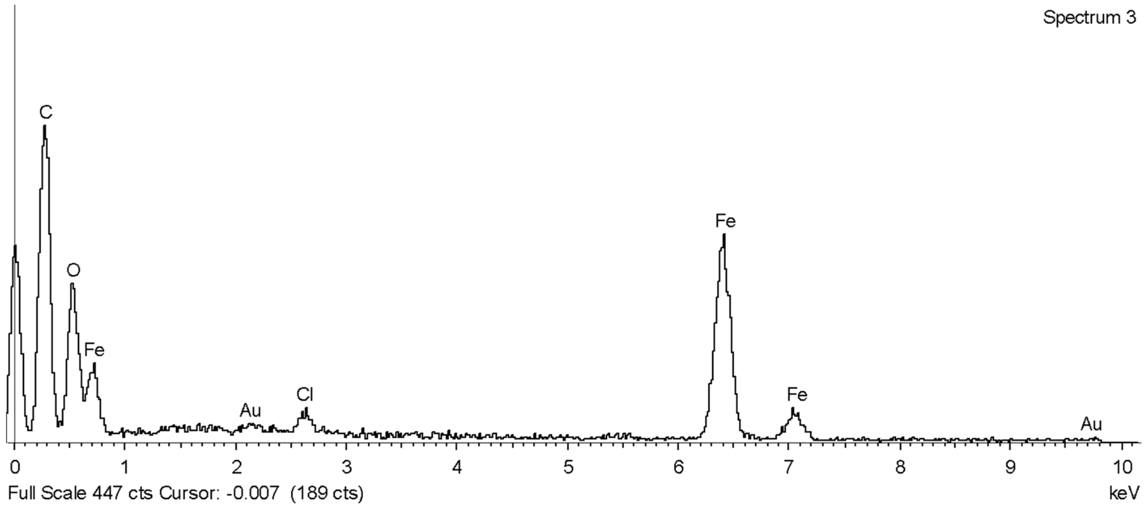
Spectrum 3
C
O
Fe
Au
Cl
Fe
Fe
Au
0 1 2 3 4 5 6 7 8 9 10
Full Scale 447 cts Cursor: -0.007 (189 cts)
keV

(c)

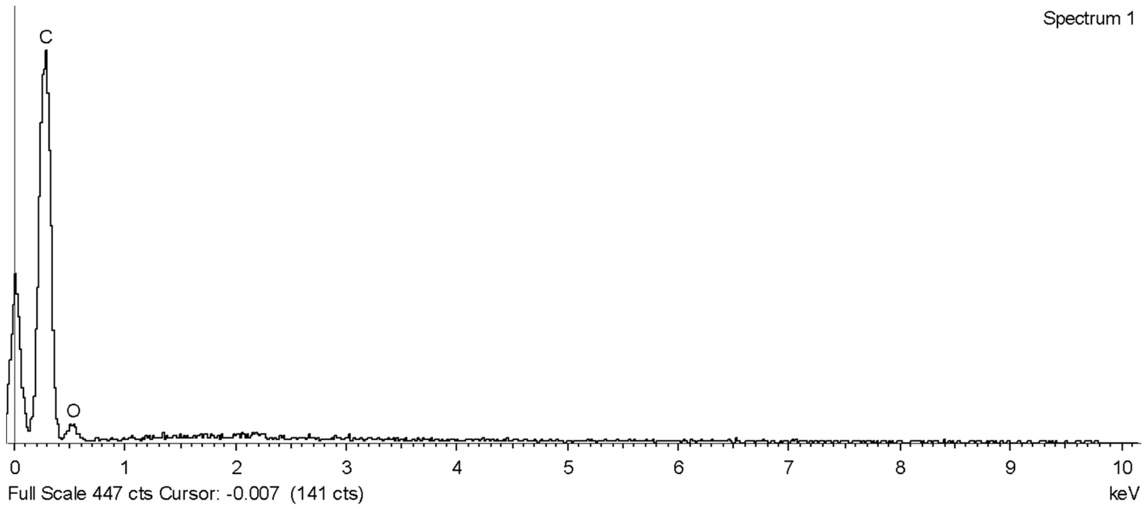
Spectrum 1
C
O
0 1 2 3 4 5 6 7 8 9 10
Full Scale 447 cts Cursor: -0.007 (141 cts)
keV

(d)

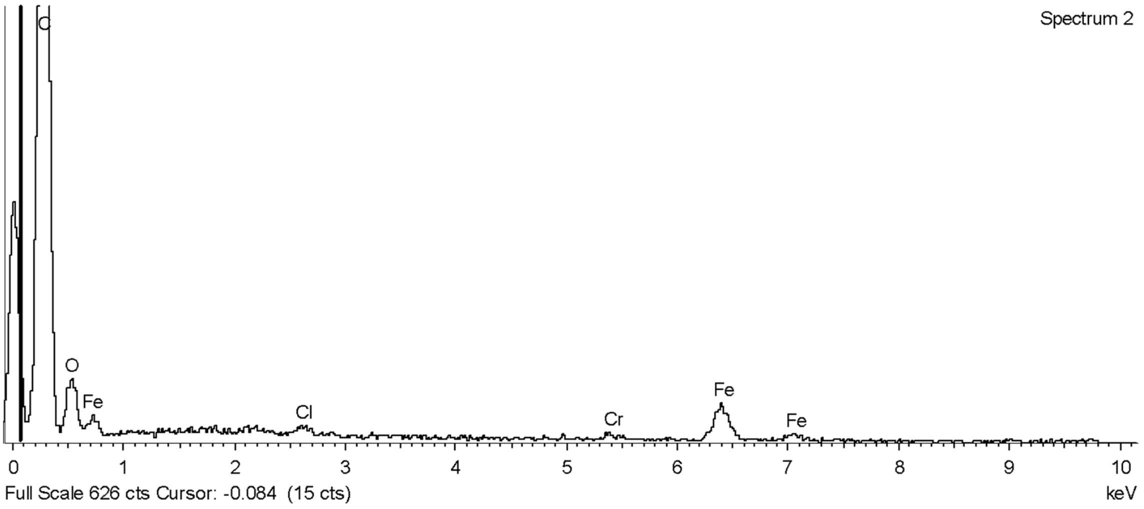

(e)

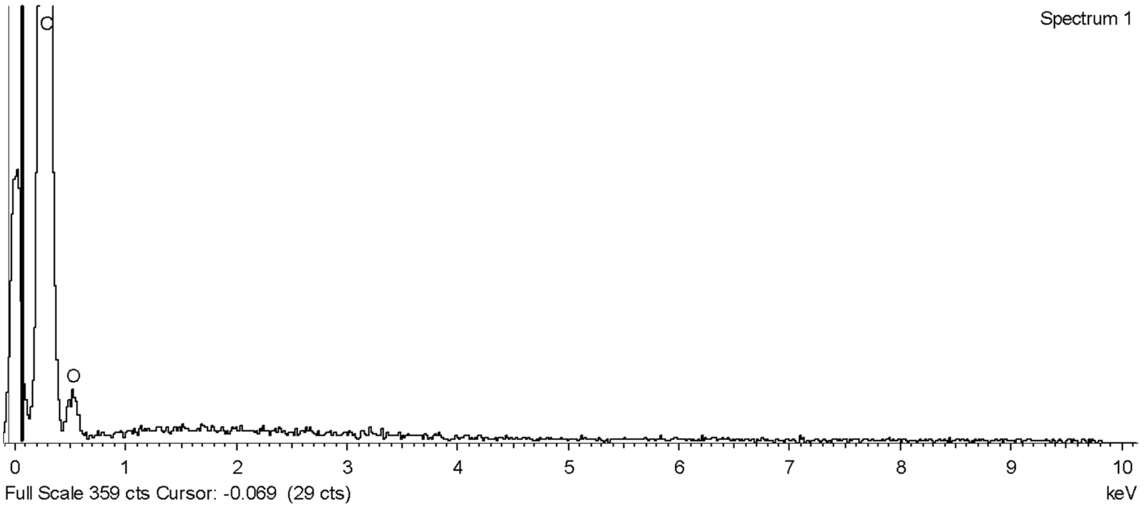

Figure S1. – EDS spectra (YY axis: intensity) of (a) hydrotris(1*H*-pyrazol-1-yl)methane dichloro-gold(III) complex [AuCl$_2$(κ^2-Tpm)]Cl (Tpm = HCpz$_3$; pz = pyrazol-1-yl) synthesized by mechanochemistry using the planetary Emax High Energy Ball Mill; (b) the mechanochemical product from reaction of an equimolar amount of HAuCl$_4$.3H$_2$O and Tpm, using the planetary Ball Mill PM 100; pristine graphene sheets before (c) and (d) after mechanochemical treatment at Planetary Ball Mill PM 100 and (e) at Emax High Energy Ball Mill.

Figure S2 presents the ATR–FTIR spectrum of the graphene sheets. It does not show the presence of oxygen-containing groups in the initial sample. It displays the characteristic C-C and C=C stretching modes at 2158, 2027 and 1975 cm^{-1}.

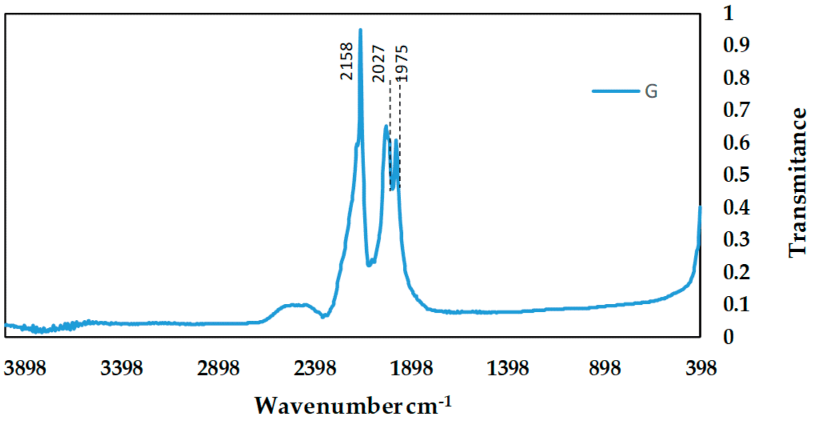

Figure S2 - ATR-FTIR spectrum of the graphene used for the immobilization of [AuCl$_2$(Tpm)]Cl_BM.

Figure S3 shows the powder X-ray diffraction patterns revealing that graphene present two peaks at 2θ = 24.6 ° and 44.6 °, which can be attributed to the amorphous graphitic carbon (002) and (100), respectively.

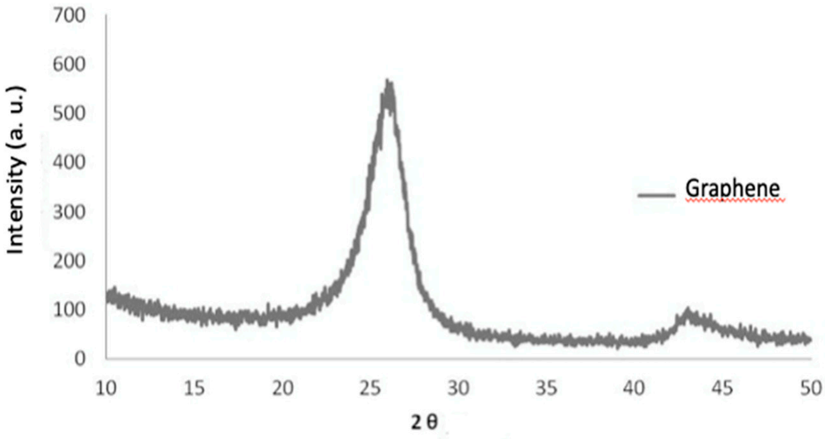

Figure S3 – PXRD of the graphene used for the immobilization of [AuCl$_2$(Tpm)]Cl_BM.
